# Observations on the Amœba Coli in Dysentery and Abscess of the Liver

**Published:** 1891-04

**Authors:** George Dock

**Affiliations:** Galveston, Texas


DANIEL'S

### Texas Medical Journal.

A Representative Organ of the Medical Profession, and an Exponent of Rational Medicine; devoted to the Organization, Advancement and Elevation of the Profession in Texas.

Published Monthly.—Subscription $2.00 a Year.

Vol. VI. AUSTIN, APRIL, 1891. No. 10.

### Original Articles.

☞CONTRIBUTED EXCLUSIVELY TO THIS JOURNAL.☜

*The Articles in this Department are accepted and published with the understanding that we are not responsible for, nor do we indorse the views and opinions of the writers by so doing.*

For Daniel's Texas Medical Journal.

### OBSERVATIONS ON THE AMŒBA COLI IN DYSENTERY AND ABSCESS OF THE LIVER.

BY GEORGE DOCK, M. D., GALVESTON, TEXAS.

[Read before the Austin District Medical Society at Austin, Texas, March 19, 1891.]

I PROPOSE to call your attention to some novel aspects presented by certain diseases which, in their ordinary forms, are well known to you—dysentery and so-called tropical abscess of the liver. This subject is always interesting since the former disease, if not the most common, is one of the most important in our latitude; whilst the latter, notwithstanding all that had been written on it, still remains one of the obscure diseases. It is at present of special interest because the researches I am about to describe show a close association, if not causal connection, of a representative of that class of living beings amongst which we have reason to believe we shall find some of the clues to the labyrinths of the etiology of infectious disease.

At the lower end of the zoological scale are the protozoa, innumerable organisms whose structure, as shown by our most searching means of investigation, is of the utmost simplicity, but whose vital phenomena are often so complicated and at all times so recondite, that our knowledge, even of the commonest, is but imperfect and fragmentary. Many of these organisms, whole classes, in fact, are obligatory parasites, living in the body cavities or even in the cells of other animals or of plants. Among these minute animals we find the causes of many destructive animal and vegetable diseases, suffice it to mention malaria and the silk-worm disease and merely to allude to the diseases, often epidemic, of many esculents.

Amongst the most common, most simply formed, but not the most thoroughly understood of the protozoa, are the amœbæ; minute specks of living matter in which we can see little more than nucleus and contractile protoplasm. These animals acquire a new importance and demand further study, in view of the investigation I am about to describe.

The etiology of dysentery has remained an enigma to the present day. Many bacteria have been looked on by their discoverers as the causes, but none of them are well attested. This is an additional reason why the observations that follow have so great an attraction.

Aside from an observation by Lambl, quoted by Nothnagel and Leuckart, amœbæ as parasites of the large intestine were first discovered by Loesch, in 1873 (Virch. Arch. 65), and named by him amœba coli. The organisms were found in the feces of a patient with chronic dysentery. Improvement of the patient's condition coincided with the decrease and final disappearance of the organisms from the stools. Loesch injected fecal matter containing amœbæ into the intestines of four dgos. On three there was no effect, but in the fourth vomiting and diarrhoea ensued, with speedy recovery, but the feces contained bloody mucus in which numbers of amoebae were found. The dog was killed on the eighteenth day. Its intestine was found inflamed, covered with bloody mucus and showing three superficial ulcers. In the mucus and in the floors of the ulcers were numerous amoebae.

Loesch thought himself justified in assuming that the amoebae irritate the intestinal mucous membrane and so hinder the healing of the ulcers.

According to Leuckart amoebae were found in the intestine of a child by Sonsino of Cairo. Grassi and Perroncito (Gazz. med. ital.—Lomb, 1879) found similar organisms in feces, but attributed no importance to them. It is not surprising to learn that Koch found amoebae in sections of the intestines from cases of dysentery, while investigating cholera in Egypt, but having his special work in hand he did not follow up the subject.

Since 1885 Dr. Kartulis of Alexandria, has published a number of observations, most of them in Virchow's Archiv. (Bd. 99 *et seq.*) as the result of which he came to the conclusion that the parasite under consideration is the cause of tropical dysentery. This belief was based on the fact that he found the amoebae in the stools of more than 500 cases of dysentery (by 1889), but failed to find them in other ulcerative intestinal diseases (enteric fever, tuberculosis). The parasites were found not only in the mucus, but also in the ulcers and in the deeper layers of the gut in fatal cases in which autopsies were obtained. In cicatrized ulcers the amoebae were not found. Kartulis described the amoebae as having all the characteristics of the common form. They measured from 12 to 30 micro-millimeters (those of Loesch 20-35), in diameter. Culture experiments were negative, as were attempts to infect guinea pigs, rabbits, dogs and monkeys.

Kartulis has also investigated the tropical abscess of the liver, in which he early found the amoebae. He divides these abscesses into two classes, excluding the pyaemic variety, which he calls idiopathic and dysenteric, the latter forming 55-60 per cent. of all cases. In the former various species of pyogenic bacteria are met with. In the latter amoebae were found in all (22) cases, sometimes with pyogenic bacteria and in one case proteus vulgaris. Kartulis explains the pathogenesis of the dysenteric liver abscess as follows: "In the ulcers of dysentery the amoebae often invade all the layers of the gut, especially the capillaries of the submucosa. From there they wander through the portal vein into the liver, accompanied by detritus, blood corpuscles and various bacteria. The latter cause suppuration, but only in consequence of the local predisposition set up by the amoebae, for these, by means of lacerations of blood vessels, often cause important lesions. That the amoebae are of great importance in the production of liver abscess is shown by the fact that in Egypt, as elsewhere, typhoid and tuberculosis ulcerations of the intestine are seldom accompanied by abscess of the liver."

Kartulis has seen two cases of liver abscess with intestinal tuberculosis, and in these he found amoebae in the pus.

It would take us too far to follow Kartulis' description of the histology of liver abscess, which, aside from the part played by the amoebae, contains nothing new.

Kartulis has also found amoebae in the stools of two cases of dysentery in Athens (Ctlb. f. Bakt. u. Parasitenk. i. 90. 54), thus indicating a new area of distribution of these organisms.

An important contribution to the subject is furnished by Massiutin, from the clinic of Professor Loesch, in Kief. (Ctlb. f. Bakt., etc., ii., 1889, 451.) Massiutin found amœbæ in five cases, only one of which had the symptoms and stools of dysentery, the others being acute or chronic cases of intestinal catarrh without bloody stools. The organisms resembled in every particular those described by the authors already quoted. Massiutin combats Kartulis' view that the amœbæ cause the "bloody diarrhœa, as they are found in other intestinal diseases." He holds that the parasites reach the intestine through drinking-water and settle specially in the mucous membrane of the large intestine, provided they find the conditions necessary for their existence. He says, however, that "the intensity of the disease called forth by them depends on the number of the amœbæ."

In the abstract of the Russian observer's article, there are evident contradictions, which are the less important at present since the whole subject is still in the stage of inquiry. Kartulis very properly takes the ground that Massiutin is not justified in asserting that his cases were not dysenteric, but on the other hand it is too early to consider them to be such on the ground of finding amœbæ in them.

The next report on these organisms, and the first in the new world, was made by Dr. Wm. Osler (Johns Hopkins Hospital Bulletin, May, 1890). The patient was a physician who had acquired a chronic dysentery at Panama, and, returning to Baltimore, developed signs of abscess of the liver. The liver was aspirated and incised, and two large abscesses found in the right lobe. In the thick, creamy pus Dr. Osler found amœbæ in large numbers, and the organisms could be found in the pus removed at the dressings up to the time of the patient's death, two weeks later. Diarrhœa, with muscus, pus, blood and sloughs in the stools, was a marked symptom in this case, and the amœbæ were found in variable numbers in the different components of the stools. Dr. Osler's full description of the organisms proves them to have been similar to those described by other observers, though not reaching the maximum size.

Afterwards Dr. Osler and his assistants found amœbæ in several cases of dysentery and liver abscess. One of the most interesting, and practically most important, is the following, reported by Dr. Simon (Johns Hopkins Hospital Bulletin, Nov., '90):

The patient was a man of 29 years, who had been treated in the Philadelphia Hospital for pleurisy, on account of a pain in the side, and cough. Later he had bloody expectoration. Occasionally there was a little blood in the stools, but no diarrhœa. In September he entered the Johns Hopkins Hostital. He had then cough, and expectoration of bloody mucus. There were two or three small stools daily, containing mucus and blood. There were no chills, but fever, and at times sweats. On the day of admission, the expectoration was noticed to be of a peculiar rusty, reddish-brown color, and purulent. Actively moving amœbæ were found in it, a fact which at once called attention to the bowels and liver. After admission there was no diarrhoea, but usually one or two well-formed stools, in which were traces of blood. Amœbæ were found in these, in great numbers. The liver was enlarged posteriorly: There was dullness over the right lung, behind, but no sign of cavity. Beside amœbæ, the sputa contained blood, alveolar epithelium, and elastic tissue; no tubercle bacilli. Cough was the only symptom.

Several cases of dysentery with amœbæ in the stools have been reported by Dr. Stengel (Medical News, November 15, '90) and Dr. J. H. Musser (University Medical Magazine, December, 1890), thus completing the not very extensive literature of the subject up to this time.

My own experience with the amœbæ coli includes twelve cases, brief histories of which I will let precede further remarks on the subject.

I. Man of 22 years, living in Galveston. Sick for three weeks, with continued fever, clouded intellect, and pain in the right iliac fossa. Stools loose at times, but not diarrhœal; no tenesmus or colic, and no blood. The case was considered one of typhoid fever by several physicians who saw the patient, until a few days before death. At that time, dullness was found at the base of the right lung. An exploring needle showed the presence of serous fluid. Fluid was also detected in the abdomen, so that it was thought there might be tuberculosis of the serous membranes. A day or two later, July 19, 1890, the patient died, and I was asked to make an autopsy, which was limited to the abdominal cavity.

The peritoneal cavity contained about two quarts of turbid serum with fibrin flakes. Between the liver and diaphragm was a large amount of bloody serum. The exploring needle had entered this. The right lobe of the liver was much enlarged, and adherent to the lateral abdominal wall. On loosening up the adhesions, I broke into an abscess cavity the size of a child's head, containing a pinkish pappy fluid with tags of necrotic tissue, some of large size, and of the most nauseating odor. In the left lobe was a necrotic focus the size of a cherry. The walls of the abscess were soft and ragged. From the ileo-caecal valve to the sigmoid flexure the walls of the colon were thick and gelatenous looking, the mucous membrane from black to deep red in color, and showing in the cœcum, when the process was most advanced, linear and follicular erosions and ulcers.

In the mucus from the colon and in the pappy fluid from the liver abscesses, large numbers of amœbae were found, but none were found in the softening focus of the left lobe.

II. R. H., age 42, came from Temple, Texas, and was admitted to St. Mary's Infirmary July 16, 1890. He said he had been sick two weeks, with fever and diarrhœa. The stools were not examined carefully, but contained mucus and blood, and there was tenesmus at times, as well as colic. While in hospital the patient was in the typhoid state. Towards the end, consolidation of the lower right lobe of the lung became apparent, with enlarged liver dullness, and pain over the latter organ. The patient sank rapidly, and died July 25, 1890, and I made the autopsy at the request of the house surgeon, six hours later.

There was general peritonitis with sero-fibrinous excudate; inflammation of the colon from the caecum down, with large ragged ulcers; liver enlarged, pale yellow, and with an abccess the size of a fœtal head at term in the right lobe. The cavity contained dirty yellow matter of creamy consistency, and rather penetrating odor. There was purulent pneumonia of the lower lobe of the right lung. The other organs showed no special changes. Specimens of pus from the liver and contents of the colon were placed in clean, dry bottles, and removed for examination, but owing to the circumstances under which the autopsy was made, no other material was obtained.

The matter from the liver, examined microscopically, showed a few broken down and macerated pus corpuscles, occasionally a red blood corpuscle. The bulk of it was made up of granular detritus, cocci, long and thick bacilli, and amœbæ. The latter were present in enormous numbers, so that from ten to fifteen could be seen in a single field of the Zeiss DD. In the contents of the intestines the organisms were found, but not in such large numbers.

III. A. A., aged 31. In Galveston six weeks. Before that, while living on the main land, had malarial fever. Three weeks before admission, (Oct. 29, 1890), diarrhœa began. The stools, at first, were hard and passed with difficulty, and accompanied by blood. On admission the stools averaged two to three a day, with tenesmus and pain in the hypochondria. The dejections were characteristically dysenteric, with mucus, blood, pus and sloughs, having very little odor. Amœbæ were found in large numbers during a period of two weeks, including a relapse after normal stools had appeared. The relapse was preceded by the reappearance of amœba in a small amount of pus adherent to the feces.

IV. A. D., aged 25. In this case the diagnosis of abscess of the liver was made by Dr. G. S. Sykes two months before the patient gave up work to undergo operation. Dr. S. could obtain no history of dysentery. After finding amœbæ as described below I examined the man carefully, but obtain no evidence of dysentery, diarrhœa, or other relevant symptom.

Dr. C. H. Wilkinson operated on the abscess November 1, '80, by resecting the eighth rib in the anterior axillary line. In the I reddish-yellow purulent matter and sloughs evacuated (2-3 pts). found amœbæ, as also in the pus removed at the dressings, but in the latter examinations the organisms were motionless, no doubt killed by the iodoform used in the tampons. The man died suddenly on the third day after operating, when an unexpected condition was found.

The course of the colon was M-shaped, the cæcum was deep and capacious, forming a pouch. On opening it its mucous membrane was found red and swollen and having numerous ulcers, from the size of a pin's head to some having a diameter of ½ by ¾ inches. They were almost all down to the muscularis, their walls slightly infiltrated. There were no ulcers, except in the cæcum, and redness and swelling extended but a short distance beyond. The abscess of the liver was small, its walls thick and dense, having had a tendency, no doubt, to healing. The cæcal ulcers contained active amœbæ and cercomonas. As causes determining death I found chronic parenchymatous nephritis, fibroid heart and retraction of the aortic valve.

V. and VII. Two cases of dysentery with classical symptoms and characteristic stools, in St. Mary's infirmary, in both of which amœba were found.

VI. F. P., aged 52. Dysentery began in San Antonio in August and continued to the time of admission to the Sealy hospital, November 22, '90. The patient had an uncontrolable desire to defecate from twenty to forty times a day. The evacuations were always bloody, sometimes looking like pure blood, at others resembling coffee grounds, or tobacco juice. Tenesmus was intense, sometimes lasting five to six minutes, without an evacuation. There was pain and tenderness on both sides of the abdomen; the general condition was verybad. In the bloody mucus amœba were found, as also *cercomonas*. A certain amount of improvement followed the treatment in this case, though amœbæ could almost always be found. Albuminuria with casts, with œdema of the extremities and lungs, however, developed, and the patient died about a month after admission. An autopsy could not be obtained, but some time later the body turned up in the dissecting room. Owing to the imperfect injection the body was very much decomposed. The liver came into my hands later. It contained a large abscess, with thin necrotic walls. The pus was entirely disorganized, so that no histological elements, or amœbæ, if present, conld be seen. By some mistake the intestines were removed from the body without inspection.

VIII. A. B., aged 42. This patient was admitted to the Sealy hospital December 2, '90, in a comatose condition, passing bloody stools involuntarily. No satisfactory history of his case could be made out. He died December 6, '90. In addition to various lesions of other viscera the colon showed exquisite dysenteric ulcers from the valve to the upper end of the sigmoid flexure. In the scrapings from the ulcers amœbæ were found in large numbers.

IX. Man of about fifty years, admitted to St. Mary's infirmary in delirium, became comatose and died, December 13, '90, before the case could be made out. Our interest in it lies in the fact that at the autopsy I found ulcerative colitis from the ileocæcal valve to the middle of the descending colon, most intense at the lower part. Amœbæ were found in the mucus and sloughs.

X. J. M., aged 41; returned from Nicaragua three months ago, where he had malarial fever, of which the blood examination shows no trace. Two months before admission (January 28, 1891) diarrhœa came on suddenly at night, with colic, frequent bloody stools, tenesmus, and rapid loss of strength. This condition ameliorated before admission, and he had partially formed stools. In one of them I found a small mass made up of pus and sloughs and containing amœbæ. They were found for several days, until the patient left the hospital, feeling well.

XI. Young man, in Sealy hospital, with two to three formed stools a day; pains in left side, but no colic or tenesmus. In a stool just as he was about to leave the hospital, believing himself well, I found the dejections formed and of natural color, but covered with a thin layer of blood, with a little pus. Under the miscroscope I found, beside pus and small sloughs, a number of active amœbæ.

XII. A patient of Dr. Hadra. Young man with diarrhœa, though without colic or tenesmus; with enlarged liver, and pain in abdomen. In a characteristic stool I found blood clots, rose-colored mucus, pus and sloughs,—some of considerable size,—and amœbæ. Dr. Hadra wished to explore the liver, but as the patient declined, refused to treat him, and I lost sight of him.

These include all the cases of dysentery I have seen since beginning the investigation. In the same period of time I have examined stools from all the other cases of diarrhœa, including several of enteric fever (two with autopsies), to which I had access. In none of them did I find amœbæ. Much as the cases cited vary in detail, they have two things in common,—ulceration in the large intestine and the amœbæ coli in the dejections. My experience, therefore, leads me in the direction of Kartulis' views. But, in the entire absence of analogies for such views, and the impossibility of using the experimental test—from our inability to isolate the organisms—we need a greater amount of testimony by far than has yet been brought forward, not only in regard to the presence of the amœbæ in certain forms of disease, but also to their absence in other processes.

All the observations cited indicate the necessity of new studies in the etiology of intestinal diseases; studies in which the amœbæ coli cannot be ignored.

Leaving this part of the subject let me call attention to some practical points brought out by these observations. In speaking of dysentery, we are too apt to forget that it is a clinical expression, and without an invariable anatomical basis. The fact that ulcerative diseases may exist in the intestine without the usual symptoms, has long been known, but is not sufficiently borne in mind. It appears from my histories that in only six cases was there a history of dysentery. In two more it might have been elicited by careful investigation, but in four it was absent. In two of these autopsies showed that the lesions were limited to the caecum. At present no general diagnostic point can be made from this, but experience in other cases makes it very clear that had the stools been examined in these and the amœbae the result would have been at least not unexpected.

Cases I and II bring up an interesting point in regard to abcess of the liver. Frerichs held that typhoid symptoms do not occur in cases of tropical abscess. But a pupil of Frerichs', Dr. B. E. Hadra, called attention to the fact that we may have such symptoms with the large abscesses complicating dysentery in Texas, in the following words (*Transactions Texas State Medical Association, 1878*): "The acute form very often presents, in the first stage, the aspect of typhoid fever. There is restlessness, low delerium, increase of temperature, diarrhœa, puffiness of the bowels, etc. As in many cases the stools contain no blood at all, as the stools in other cases often resemble the pea soup defecations known as typhoid stools, an error is easily made."

So far the discovery of the amœbae has not cleared up our knowledge of the pathology of liver abscess. In the tropical abscess necrotic softening often preponderates over suppuration, and it may be that this is determined by the amœbae, either directly or indirectly by their effect on the bacterial organisms which accompany them, a supposition which can be tested by culture experiments. But all large abscesses of the liver do not contain amœbae, as Kartulis has shown. I have examined the contents of an abscess aspirated by Dr. Randall in the case of a woman with no history except that of the abscess. A wash-basin full of brick-dust colored creamy fluid was withdrawn, in which I could find nothing but well-preserved pus corpuscles, many haematoidin crystals, and granular debris.

Enough has been said to show that both for practical purposes, as well as etiological study, the amœbae coli is of great importance. A few words on the means of recognizing it will therefore not be superfluous.

In the examination of faeces it is better to have them as recent as possible, and at the body temperature, but this is by no means absolutely necessary. In warm weather the organisms remain active even after twenty-four hours' removal from the body, and in cold weather the simple expedient of warming the slide over an alcohol lamp, is all that is necessary to bring out active motion.

The amœbae may be met with in all parts of the dysenteric stool. Most favorable to their discovery are small particles of pus, blood-stained mucus, or sloughs, and examinations of all of these are very instructive in many respects not necessary to discuss at present. Lowering a cover-glass on the drop selected, without much pressure, it should be examined by a moderately high power, about 400 diameters. If not in motion the organisms may be mistaken for degenerated cells or even starch granules, depending on the quantity of granular matter they contain. With a little experience, however, the larger ones can be recognized by their peculiar appearance, and on watching them a few moments the characteristic motion will show itself. In size the amœbae vary greatly. I have made many measurements, using a Zeiss DD with No. 3 micrometer eye-piece, and calculating the results from an object micrometer ruled in $\frac{1}{100}$ of a millimeter. The smallest I could recognize with certainty measured 13, the largest 37 micro-millimeters in diameter. Usually they are from 18 to 25, and those over 30 exceptional. In all the fine granular endosare, and the homogeneous ectosare can be made out, the former often filled with granules of variable size. In perhaps one-half a spherical, highly-refracting nucleus can be seen, lying in a clear vesicle. They also contain from one to many vacuoles, seldom a pulsating vesicle.

The inclusion of red blood corpuscles, leucocytes, bacteria and detritus granules is very common and can often be followed under the microscope. This, and the size of the larger ones, serve to distinguish them from degenerated epithelial cells, to which they have a certain resemblance. The latter, however, have larger and more distinct nuclei, and never, so far as I have seen, exhibit amoeboid motion in the stools. In their behavior towards acids and alkalis, iodine, etc., the amoebae coli do not differ from the common forms, so that detailed descriptions are unnecessary. Staining of the organisms is not very successful.

I made a number of attempts at cultivating the amoebae, in mutton broth, rice soup and liquid feces, but they always died in a few days, sometimes assuming an encysted appearance, with many vacuoles.

As to the source of these parasites we can only conjecture. The fact that several of the cases acquired their disease in Gaiveston, the water supply of which is all from overhead or underground tanks which collect from roofs, is of course an interesting one. I have made a large number of examinations of the sediment left on filters formed by tying towels over the faucets of three of these tanks and leaving them on for from an hour to two days at a time. Two of the tanks were overhead cisterns in the hospital grounds, one of which furnished drinking water, the other a wooden tank in the built-up part of the city. In all the filters I found the same things—low fungi, confervae, desmids, and a small paramecium, but no amoebae.

It will be remembered that in two of my cases cercomonas intestinalis was also found. Numbers of this infusorium were met with in the pea soup stools of a case of enteric fever (autopsy), but it, too, was absent from the drinking water examined.

From the testimony now before us we may conclude:

1. The amoeba coli is a parasite whose habitat is probably not circumscribed, having been found in Africa, Europe and North and South America.

2. It is probably a true amoeba.

3. It accompanies certain forms of ulcerative disease of the large intestine.

4. It is found in some cases in the abscess of the liver complicating those diseases.

5. We have no reason for ascribing pathogenic powers to the parasite, though the evidence is such that it offers a promising field for future investigations.

6. It probably has a certain value in the diagnosis of ulcerative diseases of the colon, and also as an indication of the efficacy of treatment.

